# Origin of marine planktonic cyanobacteria

**DOI:** 10.1038/srep17418

**Published:** 2015-12-01

**Authors:** Patricia Sánchez-Baracaldo

**Affiliations:** 1 School of Geographical Sciences, University of Bristol, Bristol BS8 1SS, UK

## Abstract

Marine planktonic cyanobacteria contributed to the widespread oxygenation of the oceans towards the end of the Pre-Cambrian and their evolutionary origin represents a key transition in the geochemical evolution of the Earth surface. Little is known, however, about the evolutionary events that led to the appearance of marine planktonic cyanobacteria. I present here phylogenomic (135 proteins and two ribosomal RNAs), Bayesian relaxed molecular clock (18 proteins, SSU and LSU) and Bayesian stochastic character mapping analyses from 131 cyanobacteria genomes with the aim to unravel key evolutionary steps involved in the origin of marine planktonic cyanobacteria. While filamentous cell types evolved early on at around 2,600–2,300 Mya and likely dominated microbial mats in benthic environments for most of the Proterozoic (2,500–542 Mya), marine planktonic cyanobacteria evolved towards the end of the Proterozoic and early Phanerozoic. Crown groups of modern terrestrial and/or benthic coastal cyanobacteria appeared during the late Paleoproterozoic to early Mesoproterozoic. Decrease in cell diameter and loss of filamentous forms contributed to the evolution of unicellular planktonic lineages during the middle of the Mesoproterozoic (1,600–1,000 Mya) in freshwater environments. This study shows that marine planktonic cyanobacteria evolved from benthic marine and some diverged from freshwater ancestors during the Neoproterozoic (1,000–542 Mya).

Cyanobacteria have fundamentally transformed the geochemistry^1^,^2^ of our planet. Multiple lines of geochemical evidence support the occurrence of intervals of profound global environmental change at the beginning and end of the Proterozoic (2,500–542 Mya)^3^,^4^,^5^. While it is widely accepted that the presence of molecular oxygen in the early fossil record was the result of cyanobacteria activity, little is known about how cyanobacteria evolution (e.g., habitat preference) may have contributed to changes in biogeochemical cycles through Earth history. Geochemical evidence has indicated that there was a first step-increase in the oxygenation of the Earth’s surface, which is known as the Great Oxidation Event (GOE), in the early Paleoproterozoic (2,500–1,600 Mya)^1^,^2^. A second but much steeper increase in oxygen levels, known as the Neoproterozoic Oxygenation Event (NOE)^4^,^6^,^7^, occurred at around 800 to 500 Mya^5^,^8^. Recent chromium (Cr) isotope data point to low levels of atmospheric oxygen in the Earth’s surface during the mid-Proterozoic^3^, which is consistent with the late evolution of marine planktonic cyanobacteria during the Cryogenian^9^; both types of evidence help explain the late emergence and diversification of metazoans^10^.

Understanding the evolution of planktonic cyanobacteria is an essential question because their origin fundamentally transformed the nitrogen and carbon cycles towards the end of the Pre-Cambrian^9^. It remains unclear, however, what evolutionary events led to the emergence of open-ocean planktonic forms within cyanobacteria, and how these events relate to geochemical evidence during the Pre-Cambrian^4^. So far, it seems that ocean geochemistry (e.g., euxinic conditions during the early- to mid-Proterozoic)^4^,^7^,^11^ and nutrient availability^12^ likely contributed to the apparent delay in diversification and widespread colonization of open ocean environments by planktonic cyanobacteria during the Neoproterozoic^9^.

Marine phytoplankton today contribute to almost half of the Earth’s total primary production^13^. Within the cyanobacteria, only a few lineages colonized the open-ocean (i.e., *Crocosphaera* and relatives, cyanobacterium UCYN-A, *Trichodesmium*, as well as *Prochlorococcus* and *Synechococcus*)^14^,^15^,^16^,^17^. From these lineages, N-fixing cyanobacteria are particularly important because they exert a control on primary productivity and the export of organic carbon to the deep ocean^14^, by converting nitrogen gas (N_2_) into ammonium (NH_4_^+^), which is later used to make amino acids and proteins. Marine picocyanobacteria (i.e., *Prochlorococcus* and *Synechococcus*) numerically dominate most phytoplankton assemblages in modern oceans contributing importantly to primary productivity^16^,^17^,^18^. While some planktonic cyanobacteria are unicellular and free living cells (e.g., *Crocosphaera, Prochlorococcus, Synechococcus*), others have established symbiotic relationships with prymnesiophyte algae^15^. Amongst the filamentous forms, *Trichodesmium* are free-living and form aggregates. However, filamentous heterocyst-forming cyanobacteria (e.g., *Richelia*, *Calothrix*) are found in association with diatoms such as *Hemiaulus, Rhizosolenia*, and *Chaetoceros*^19^,^20^,^21^.

While environmental conditions might have prevented the widespread diversification of planktonic forms during most of the Pre-Cambrian^9^, the evolutionary history of marine planktonic cyanobacteria (e.g., habitat preferences, morphology) likely played an important role in the events surrounding the emergence of complex life in the oceans. Data from 131 cyanobacterial genomes was used to carry out large-scale multi-gene analyses of cyanobacteria; these analyses provide robust evidence for the early evolution of filamentous forms and mat-forming/benthic cyanobacteria and a delay in the emergence of marine planktonic cyanobacteria towards the end of the Pre-Cambrian. Two separate data sets (protein and nucleotide sequence data) and five different types of substitution models (including the CAT-GTR model) were used to explore the timing of key evolutionary events that led to the late emergence of planktonic cyanobacteria. Bayesian stochastic character mapping analyses were performed to study the evolutionary traits involved in the emergence of marine planktonic cyanobacteria such as loss of filamentous forms (and presumably intracellular communication), decrease in cell diameter, and shifts in habitat preference within cyanobacteria. This study also shows that marine planktonic cyanobacteria evolved from benthic marine and freshwater ancestors.

## Results

### Phylogenetic relationships

**Figure 1:**
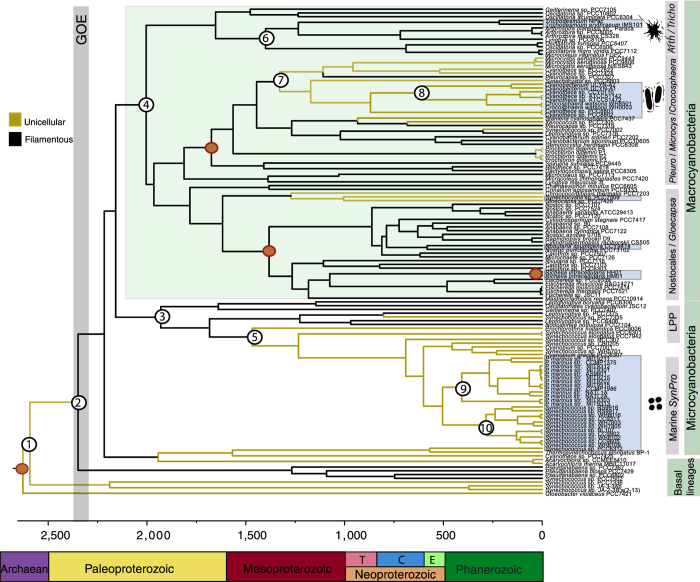
The origin and diversification of cyanobacteria as inferred from geologic time. The phylogenetic tree was estimated based on 135 proteins and two ribosomal RNAs (SSU and LSU) from 131 taxa implementing Maximum Likelihood in RAxML GUI v.1.1^61^. Bayesian relaxed molecular clock analyses were carried out in MCMCtree^31^. For the tree shown age estimates were estimated under the independent rates model^33^ for the RNA data set. Four calibrations (brown circles) were used^2^,^67^,^68^,^69^ for the tree shown and were treated as soft bounds. The root of the tree was set with a maximum age of 2,700 Myr^63^ and a minimum age of 2,320 Myr^2^. Numbered nodes 1–10 indicate divergence times for clades and key evolutionary events in the evolution of cyanobacteria including: the first origin of filamentous cells, ancestors of the Microcyanobacteria and Macrocyanobacteria, unicellular N-fixers and the marine *Synechococcus* and *Prochlorococcus* clades. Age estimates are given in Table 1, which includes the corresponding values for the posterior 95% confidence intervals.

An increase in genome sequencing and taxon-sampling have allowed for broad coverage of a range of morphologies, lifestyles, and metabolisms within cyanobacteria^22^. The analyses performed here included a large phylogenetic data set consisting of 131 genome taxa with a total of 56,251 amino acids (aa) and 4,555 base pairs (bp). Whilst analyses have recovered well-supported monophyletic groups previously reported^9^,^22^,^23^,^24^,^25^,^26^, new genomic data have revealed novel deep-branching relationships of major cyanobacteria lineages^22^,^24^ (Fig. 1 and Supplementary Fig. S1). In this study *Pseudanabaena* appears as an early divergent lineage within cyanobacteria (Fig. 1 and Supplementary Fig. S1) occurring in 88% of the Maximum Likelihood trees generated for each gene alignment (137 genes) generated in SATé 2.2.3^27^. A basal position for *Pseudanabaena* is consistent with recent large-scale multi gene studies^22^,^28^. Previous studies suggesting that *Pseudanabaena* is a derived lineage were based on SSU rRNA datasets^9^,^25^.

Genomic data have also clarified problematic phylogenetic relationships such as the positioning of the filamentous LPP group (Fig. 1 and Supplementary Fig. S1). New data strongly support sister relationships between the LPP clade (Supplementary Fig. S1) and *Prochlorothrix, Synechoccocous elongatus* and the *SynPro* clade (*Synechococcus, Prochlorococcus, Cyanobium*). While the inclusion of recently sequenced genomes^22^,^24^,^29^,^30^ suggest a new placement for *Trichodesmium* (Fig. 1 and Supplementary Fig. S1), more *Oscillatoria*-like genomes are needed to fully understand the placement of this important lineage. Modern marine planktonic cyanobacteria evolved within two major groups of cyanobacteria, here referred to as the Microcyanobacteria and the Macrocyanobacteria since they are well-supported monophyletic clades (Supplementary Fig. S1). Whilst the Microcyanobacteria contain lineages with smaller cell diameters (<3 μm), the Macrocyanobacteria contain lineages with larger cell diameters (>3 μm; Supplementary Fig. S3). The Macrocyanobacteria are the most taxonomically and ecologically diverse clade including lineages such as *Synechocystis, Pleurocapsa, Microcystis, Trichodesmium* and the Nostocales, amongst others (Fig. 1).

### Relaxed molecular clock analyses

Age divergences were estimated using two independent data sets, RNA (SSU and LSU: 4,555 bp) and proteins (18 genes: 4,980 aa), and applying a Bayesian approach^31^,^32^. Four calibration points were implemented, three of which have been previously used^9^,^25^. Relaxed molecular clock analyses were performed under the independent-rates model^33^, which has been shown to be the best fitting molecular clock model for cyanobacteria based on Bayes Factors^9^. Four different models of molecular evolution were implemented for proteins and RNA in MCMCtree and the CAT- GTR model for proteins and RNA in Phylobayes (Table 1). The implementation of two different maximum ages for the origin of oxygenic photosynthesis (i.e., 3,000 and 2,700 Myr) resulted in different age estimates for the origin of filamentous forms (node 2). While an older maximum age (3,000 Myr) predicts the origin of filamentous forms (node 2) before the GOE with estimates ranging between 2,665 and 2,559 Mya, a younger maximum age (2,700 Myr) predicts filamentous forms appearing around the time of the GOE between 2,460 and 2,351 Mya (Fig. 1, Table 1). Overall an older maximum age (3,000 Myr) tends to make ages older across all analyses.

Results were consistent across models of molecular evolution within each data set. There is strong evidence for a Neoproterozoic or early Cambrian origin for marine unicellular N-fixers (i.e., the *Crocosphaera* clade) and the filamentous *Nodularia spumigena* CCY9414. Age estimates appear to be younger for *Prochlorococcus* (nodes 9) and *Synechococcus* (node 10) based on the nucleotide data set, in contrast to the protein data set (Table 1). All analyses however provide robust evidence for the relatively late evolution of marine planktonic cyanobacteria. Other marine N-fixers evolved during the Phanerozoic such as *Richelia* (a diatom symbiont) and the cyanobacterium UCYN-A clade (a coccolithophore symbiont; Fig. 1). Age estimates across all analyses are summarized in Table 1 and Fig. 1, and are mostly in broad agreement.

### Bayesian trait evolution analyses

**Figure 2:**
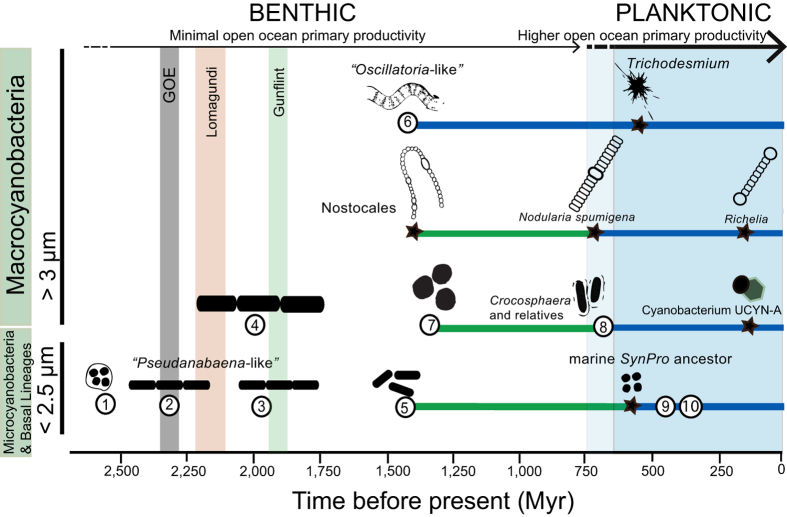
Timing and trends in cell diameter, loss of filamentous forms and habitat preference within cyanobacteria. Nodes shown (1–10) correspond to Fig. 1 and Table 1. Stars represent common ancestors that appear in Fig. 1 but ages are not given in Table 1. The timing of the Great Oxidation Event (GOE) is after ref. 2, the Lomagundi-Jatuli Excursion after ref. 55 and Gunflint formation after ref. 73. Green lines represent freshwater lineages and blue lines represent marine lineages based on Bayesian inference of character evolution (stochastic character mapping analyses; Supplementary Fig. S4). Cartoons are not drawn according to scale; taxa with smaller cell diameter are shown at the bottom and larger cell diameter at the top.

The earliest cyanobacteria were likely unicellular (node 1) and inhabited low salinity environments (Fig. 2 and Supplementary Fig. S4). Living relatives of these early divergent lineages have been isolated from terrestrial/freshwater environments (e.g., *Pseudanabaena* PCC6802 *Cyanothece* PCC7425, Fig. 1), hot-springs (e.g., *Thermosynechococcus elongatus* BP-1) and coastal marine habitats (e.g., *Acaryochloris* and *Synechococcus* PCC7336). Bayesian stochastic character mapping analyses revealed that filamentous cyanobacteria evolved early on and different molecular clock analyses indicate filamentous forms evolved around 2,665 to 2,351 Mya and the GOE (node 2; Figs 1 and Supplementary Fig. S2). Ancestors of early filamentous forms likely resembled modern relatives of *Pseudanabaena* and the LPP clade (nodes 2 and 3; Fig. 2). All Basal Lineages and the Microcyanobacteria have retained small cell diameters exhibiting cells that are less than 3 μm, with most lineages exhibiting diameters that are less than 2 μm (Supplementary Fig. S3 and Supplementary Table S2). Interestingly, further decrease in cell diameter characterizes the evolution of the marine *Prochlorococcus* within the *SynPro* clade^26^. Also a switch from filamentous to unicellular cell types occurred (node 5; Figs 1 and 2) around 1,994 to 1,421 Mya (Table 1).

All analyses suggest that the Macrocyanobacteria clade, exhibiting larger cell diameters (>3 up to 50 μm), may have evolved just after the GOE with age estimates ranging between 2,386 and 1,894 Mya (node 4; Fig. 1 and Table 1 and Supplementary Fig. S3). Within this clade two opposite evolutionary trends were found: 1) an increase in cell diameter (e.g., *Fischerella* and *Mastigocladopsis*) within the Nostocales, and 2) a decrease in cell diameter (node 7) within the clade containing *Microcystis* and *Crocosphaera* relatives (Supplementary Table S2, Supplementary Fig. S3). A switch from filamentous to unicellular forms also occurred (node 7, Fig. 1 and Supplementary Fig. S2) around 1,437 to 1,047 Mya in freshwater habitats (Supplementary Fig. S4). Whilst unicellular marine N-fixing cyanobacteria (e.g., *Crocosphaera* and relatives) and *Nodularia spumigena* CCY9414 diverged from freshwater ancestors, *Trichodesmium* evolved from filamentous coastal marine lineages (Fig. 1 and Supplementary Fig. S4).

## Discussion

### Stem vs crown groups

Recent genomic data have improved the resolution of the cyanobacteria tree of life helping with the interpretation of the geological record^9^,^25^,^28^. Cyanobacteria fossils with a cell diameter bigger than 3 μm appeared in the Belcher Subgroup with fossils such as colonial coccoids (Eoentophysalis) and colonial ellipsoids (Eosynechococcus)^34^,^35^. *Oscillatoria*-like filamentous fossils (e.g., *Gunflintia*) appeared in the Gunflint iron formation^36^, and *Halythrix* sp. in the Belcher subgroup^34^. At approximately 1,900 Myr, microfossils with increased cell diameters as well as sheaths became common^35^. These findings are consistent with the evolutionary studies shown here in which ancestors with inferred cell diameters larger than 3 μm (node 4), the Macrocyanobacteria, postdate the GOE (Table 1, Fig. 2)^25^. It is therefore likely that the first appearance of reliable cyanobacteria fossils observed at around 2,000 Myr^35^,^37^ is indicative of an ancient cyanobacteria radiation^23^. Interestingly, age estimates based on molecular clock studies show a lag between the early origin of oxygenic photosynthesis^25^,^28^,^38^,^39^ and the first reliable evidence of cyanobacteria in the fossil record at around 2,000 Myr^35^,^37^,^40^. The Macrocyanobacteria clade (node 4) also evolved traits necessary for establishing thick laminated mats^23^,^25^,^26^ and have shown a significant shift in diversification rates^39^. It is not surprising that this clade contains the highest taxonomic and ecological diversity of modern cyanobacteria.

Age estimates shown here suggests that there was a delay between the appearance of the first reliable cyanobacteria fossils and the ancestors of the crown groups containing marine planktonic cyanobacteria (e.g., Nostocales/*Gloeocapsa*, *Arthrospira*/*Trichodesmium* and *Pleurocapsa*/*Microcystis*/*Crocosphaera* clades; Table 1; Fig. 1). The great majority of modern cyanobacteria can be traced back to the late Paleoproterozoic and the Mesoproterozoic, implying that older cyanobacteria fossils (e.g., *Gunflintia*) belong to stem groups with no living relatives. Crown groups with morphologies that required cell differentiation and division of labor (e.g., Nostocales) evolved during the Mesoproterozoic (Fig. 1). Cell differentiation is particularly important for some marine N-fixing planktonic cyanobacteria that evolved during the Neoproterozoic (e.g., *Nodularia spumigena*) and Cretaceous (e.g., *Richelia*). The evolution of cell differentiation mechanisms would have involved a specific program of gene expression including the induction of regulatory genes and of genes encoding the proteins for the morphological and biochemical differentiation for specialized cells (i.e., heterocyst)^26^. Comparative genomic studies have shown that more complex morphologies characteristic of crown groups (e.g., Nostocales) required the evolution of additional genes involved in signal transduction and transcription-related functional categories^22^. Genomic and trait evolution studies have also revealed that more complex morphologies within cyanobacteria exhibit bigger genome sizes^26^ presumably as a result of the more elaborate metabolic processes involved in these lineages^41^.

### A historical perspective

Previous broad taxonomic and phylogenomic studies of prokaryotes studies have inferred a terrestrial/freshwater ancestry of cyanobacteria^42^,^43^. Trait evolution studies of cyanobacteria, implementing large genomic data sets studies, have come to similar conclusions^23^,^25^,^29^. The Bayesian stochastic character mapping analyses presented here confirm that cyanobacteria first evolved in freshwaters. Moreover, most cyanobacterial lineages inhabited benthic, terrestrial and/or shelf environments for most of the Proterozoic. Interestingly, in modern habitats, benthic cyanobacteria are much more taxonomically diverse. This is perhaps due to the great variety of available niches in coastal environments (e.g., intertidal or infralittoral areas)^44^. The early establishment of mat-forming filamentous cyanobacteria (Fig. 1)^25^,^28^ and subsequent dominance of benthic microbial communities would have restricted primary productivity to terrestrial and ocean margins (Fig. 2). The small area of fresh waters and ocean margins imply that the global biogeochemical impact of oxygenic photosynthesis would have been minimal until cyanobacteria started colonizing the open ocean^9^,^25^, which currently covers approximately two-thirds of the Earth’s surface^41^. Some marine planktonic lineages had a freshwater ancestor (Fig. 2). This is illustrated by the unicellular marine clades such as *Crocosphaera* and *SynPro*. Marine lineages adapted to marine environments by acquiring the machinery that enables them to osmoregulate in marine environments such as the set of genes responsible for the synthesis of compatible solutes: glucosylglycerol (GG), glucosylglycerate (GGA) and glycine betaine (GB)^45^,^46^.

This study has revealed that decrease in cell size and a switch from filamentous to unicellular forms or loss of filamentous forms, in the lead up to the origin of the *Crocosphaera* and the *SynPro* clades, likely played a key role in the emergence of a planktonic life style. Convergent evolution with regard to the emergence of unicellular phytoplankton forms suggests similar selective pressures taking place (e.g., nutrient starvation) on the evolutionary history of these lineages. Other strategies such as gas vesicles evolved to cope with buoyancy regulation amongst some marine filamentous lineages such as *Trichodesmium* and *Nodularia spumigena*.

Marine planktonic habitats likely provided a challenging environment for cyanobacteria to proliferate into since the ocean remained anoxic for most of the Proterozoic^4^,^47^,^48^. Under anoxic conditions, including episodes of euxinia (anoxic environments with the presence of hydrogen sulphide) and ferruginous conditions (see recent reviews on ocean geochemistry of the Pre-Cambrian^4^,^11^), key trace metals essential for N-fixation would have been depleted. This is particularly the case for micronutrients such as molybdenum (Mo) an essential constituent of the nitrogenase enzyme involved in N-fixation^49^. While marine mat-forming cyanobacteria such as *Microcoleus chthonoplastes* are capable of performing sulphide-dependent anoxygenic photosynthesis, this biogeochemical process appears to serve as a detoxification mechanism^50^ in response to an inherent active sulphur cycle found in microbial mats. In microbial mats, the establishment of vertically stratified layers (known as laminated structures) allows for the spatial separation of oxygenic photosynthesis and N-fixation (an anaerobic process inhibited by oxygen)^50^. The segregation of biogeochemical processes could have persisted through most of the Pre-Cambrian, allowing the coexistence of N-fixers and oxygenic phototrophs. Interestingly, *Trichodesmium* is the only modern planktonic filamentous N-fixer that diverged from mat-forming relatives^51^.

### Marine planktonic cyanobacteria

Only a few lineages within cyanobacteria adapted to the lack of nutrients (oligotrophy) characteristic of the Earth’s open ocean. Age estimates suggest that there was an interval of more than a billion years between the timing of origin of the common ancestor of the Macrocyanobacteria and the origin of modern marine planktonic N-fixers (Fig. 1, Table 1). Nitrogen-fixing cyanobacteria evolved different morphologies and physiological strategies within the Macrocyanobacteria. At least three lineages evolved independently towards the end of the Pre-Cambrian: *Crocosphaera* and relatives, *Trichodesmium*, and *Nodularia spumigena*. Ancestors of the unicellular N-fixers, *Crocosphaera* clade, underwent decrease in cell diameters and a switch from filamentous to unicellular forms. While unicellular marine N-fixers, the *Crocosphaera* clade, diverged from freshwater relatives, *Trichodesmium* evolved from filamentous mat-forming cyanobacteria which have modern relatives found in benthic and in marine littorals^9^,^51^. Within the Nostocales, *Nodularia spumigena* CCY9414, a lineage currently found in the Baltic Sea in salty or brackish waters, diverged from freshwater relatives towards the end of the Pre-Cambrian (Fig. 1). Recent planktonic lineages such as *Richelia* and the cyanobacterium UCYN-A clade evolved as symbionts during the Cretaceous.

Decrease in cell diameter was part of the evolutionary history of the major primary producers, *Prochlorococcus* and *Synechococcus* within the Microcyanobacteria (Fig. 1). The abundant marine *Prochlorococcus* and *Synechococcus* shared a common ancestor and evolved within the Microcyanobacteria (Fig. 1). The *SynPro* clade is sister to the filamentous *Prochlorothrix* and nested within the LPP clade (Fig. 1 and Supplementary Fig. S1). This phylogenetic relationship indicates that there was a switch from filamentous to unicellular forms^39^,^52^ in which cell adhesion and intracellular communication were likely lost. Within the evolution of the marine *SynPro* clade, there is also a trend in the decrease of genome and cell size^17^,^26^. Convergent evolution with regard to a decrease in cell diameter highlights the advantage of smaller cells when inhabiting oligotrophic environments. In modern oceans small phytoplankton cells usually dominate phytoplankton communities under oligotrophic conditions, such as the oceanic gyres, whereas larger phytoplankton cells are more abundant along continental margins and in upwelling zones, where nutrient concentrations tend to be higher and more variable^53^. Divergence times (Table 1) mostly agree with a Neoproterozoic or early Phanerozoic origin for *Prochlorococcus* (node 9) and the marine *Synechococcus* (node 10).

### Lack of evidence for early marine planktonic cyanobacteria

After the GOE, it has been assumed that marine cyanobacteria were responsible for the increase in primary productivity (Lomagundi–Jatuli excursion) and carbon burial around 2,200 to 2,060 Mya^2^,^54^,^55^. Yet if marine planktonic cyanobacteria were involved in the carbon burial (e.g., Lomagundi-Jatuli excursion), evolutionary studies have provided no evidence so far supporting the survival of early marine planktonic lineages (Fig. 1 and Supplementary Fig. S4) or perhaps these lineages have not yet been discovered. While marine living planktonic cyanobacteria cannot be traced back to the early Paleoproterozoic (Figs 1 and 2), we cannot discard the possibility that early marine cyanobacteria lineages went extinct after the GOE due to changes in marine water chemistry resulting from freely available oxygen (e.g., euxinic conditions)^4^,^11^,^12^. It is clear that more efforts are needed studying early divergent lineages of cyanobacteria in order to unravel the transition from a terrestrial to marine biosphere.

## Concluding remarks

This study sheds light on the evolutionary steps that led to the recent evolution of marine planktonic cyanobacteria. Loss of filamentous forms, decrease in cell diameter, and shifts in habitat preference from freshwater to marine were involved in the emergence of planktonic forms. Most groups of contemporary cyanobacteria can be traced back to the Mid-Proterozoic. Early cyanobacteria likely inhabited benthic, terrestrial and/or coastal marine environments. The early restriction of cyanobacteria to terrestrial and costal environments, in contrast to the vast oceans, helps explain the delay in the oxidation of the Earth’s surface during the Pre-Cambrian. Primary productivity would have significantly increased once planktonic phytoplankton became prominent towards the end of the Pre-Cambrian and early Phanerozoic.

## Materials and Methods

### Alignment and taxon sampling

Alignments including 135 protein-coding genes (56,251 aa) and two ribosomal RNAs (4,555 bp) were analysed for 131 genome taxa. All sequence data for the 131 cyanobacterial genomes were obtained from GenBank (http://www.ncbi.nlm.nih.gov) and using Geneious R6. The chosen genes (135 proteins and two ribosomal RNA: SSU and LSU) were universally present in cyanobacterial taxa, evolutionarily conserved and had a minimum number of gene duplications^23^,^56^. Principal coordinates analyses were used to identify ortholog genes that belong to a conserved ‘core’ gene set in cyanobacteria^56^. For the protein data set, genes chosen represent a wide diversity of cellular functions. These functions range from Information Processing (IP; transcription, translation, DNA replication and repair), Metabolism (Met), Cellular Processes (CP; including cell division, cell envelope biogenesis, motility and secretion) and General Function Prediction (GFP). For a detailed list with names and description of the genes included in this study see Blank and Sánchez-Baracaldo^25^. Each gene was aligned independently using SATé 2.2.3^27^, a multiple sequence alignment and phylogenetic reconstruction program. Single gene alignments generated in SATé were imported into Mesquite v. 2.75^57^ to obtain ‘nexus’ and ‘phylip’ format files for subsequent analyses. Single alignments were later concatenated into a single nexus format file using Sequence Matrix v 100.0^58^. Two concatenated matrices were obtained: one for protein-coding genes and a second one for ribosomal RNAs; both of these matrices were used to estimate tree topologies as described below.

### Phylogenetic analyses

Maximum likelihood analyses were performed in RAxML 7.2.6^59^ and Phylobayes^32^. A multiple gene approach was implemented to establish the deep-branching relationships in the cyanobacterial tree. All sequence data for 131 cyanobacterial genomes were obtained from GenBank (http://www.ncbi.nlm.nih.gov) and using Geneious 6.1.4. A total of 135 protein-coding genes (with 56,251 aa) and two ribosomal RNAs (with a total of 4,555 bp) were used to establish phylogenetic relationships of cyanobacteria. ProTest v.2.4^60^ was used to estimate the best model of evolution for the protein set. To analyse the protein sequences I implemented the LG model and G (gamma-distribution with 4 rate categories). Matrices containing the protein and RNA data set were imported into RAxML GUI v.1.1^61^ and up to 50 maximum likelihood trees were generated. To obtain statistical support analyses were performed in RAxML 7.0.3^59^ (Supplementary Fig. S1). RAxML analyses recovered the same well-supported monophyletic groups previous reported by recent phylogenomic studies^22^,^23^,^25^,^28^,^62^.

### Bayesian divergence time estimation

Divergence times were estimated implementing a Bayesian relaxed molecular clock approach in MCMCTree^31^ and Phylobayes^32^ (Table 1). Two independent data sets were assembled to estimate age divergences: 1) eighteen proteins, and 2) two RNA genes (SSU and LSU). The independent-rates model was implemented as previous studies of cyanobacteria have shown that this model is favored over the auto-correlated rates model^9^. Since cyanobacteria have an ancient origin, independent rates more likely represent greater variation in inherited factors in contrast to auto-correlated rates^9^. Because the current implementation of MCMCtree and Phylobayes do not allow the use of mixed (nucleotides and amino acids) data sets. Ages were estimated using both type of data sets separately as follows: 1) Protein data set with a total of 18 proteins (AtpA, AtpB, AtpH, L1, L4, L5, NdhH, PetB, PetD, PsaA, PsbE, RbcL, S10, S13, S19, SecY, TufA and Ycf3), and 2) nucleotide data set including two RNAs (SSU and LSU). Supplementary Table S1 contains a list of gene names and substitution rates for the analyses performed in this study.

In MCMCtree, I implemented four models of nucleotide and protein evolution (See Supplementary Information on line). For all age calibrations, soft bounds were specified with 2.5% tail probabilities above/below these limits, allowing for molecular data to correct for conflicting fossil information. In Phylobayes, I implemented the CAT-GTR replacement model for both nucleotides and amino acids^32^. For all non-calibrated nodes, I used a birth-death prior on divergence times I also performed two separate experiments two independent experiments with permissive gamma distributed root priors that allowed a 95% credibility interval of the root node to range between 2,320–2,700 Mya and 2,320–3,000 Mya. See supplementary information for a more detailed description of the relaxed molecular clock analyses performed.

### Fossil constraints

Experiments reported here (Table 1) were performed implementing two maximum ages for the cyanobacterial root: 2,700 Myr^63^ and 3,000 Myr^64^. The minimum age for the cyanobacterial root was set at 2,320 Myr (the rise in atmospheric oxygen)^2^. Based on geochemical evidence a younger (2,500 Myr)^65^ age is also reported for the origin of oxygenic photosynthesis. I also calibrated the trees with fossils exhibiting unique morphological features that could be assigned to well-supported groups such as the Nostocales and the Pleurocapsales. In the Nostocales, akinetes are thick-walled dormant cells to enable a response to extreme cold and desiccation resistant environmental conditions^66^. These specialized cells are present in most species amongst the Nostocales and likely evolved once in this group. Akinetes have been shown to have a single ancestor based on trait evolution studies^23^. A maximum age of 2,100 Myr was used for this monophyletic group for which it has been hypothesised that specialised cells such as the heterocyst evolved in response to free available oxygen in the atmosphere^67^. The Pleurocapsales are characterised by having multiple fission, a unique phenotypic property that distinguishes members of this group from other cyanobacteria. The Pleurocapsales also belong to a well-supported monophyletic group (Supplementary Fig. S1) including two strains of *Pleurocapsa* (PCC 7319 and PCC 7327). A minimum age of 1,700 Myr^68^ and a maximum age of 1,900 Myr (Gunflint iron formation and the first appearance of reliable cyanobacteria fossils observed in the fossil record^35^,^37^ were implemented. Finally, a maximum age of 110 Myr was implemented for *Hemiaulus*^69^ as these organisms host the symbiont *Richelia*^19^,^20^,^21^.

### Bayesian inference of character evolution

To infer the evolution of cell type, cell diameter and habitat, I used Bayesian stochastic character mapping^70^. Analyses were implemented in SIMMAP v1.5^71^ and used relative time calibrated trees generated in MCMCtree^31^ for the protein set as described above. No prior on the rate parameter was used, as I wanted to use the branch lengths as a direct estimate of rate of evolution. A β prior is implemented in SIMMAP on the symmetry of the transition rate matrix. Cell type or morphology were coded as 0 = unicellular, and 1 = filamentous. Cell diameter data were coded as discrete characters, where 0 = average cell diameter raging from 1 to 2 μm, 1 = average cell diameter raging 2 to 3 μm, 2 = average cell diameter raging 3 to 5 μm, and 3 = average cell diameter equal to or greater than 5 μm. Habitat were coded as 0 = freshwater, and 1 = brackish, marine or hypersaline. Character states were obtained from the Bergey’s Manual of Systematic Bacteriology^72^, previous studies of trait evolution of cyanobacteria^25^, and other cyanobacteria studies^22^. For binary characters (habitat and cell type) the bias parameter was drawn from a symmetrical β prior. Since SIMMAP uses a symmetrical β prior on the symmetry of the transition rate matrix, this influences the degree to which transitions favor state 0 over 1. The shape of the β distribution is described by the α parameter and discretized into κ categories. I performed sensitivity experimenting using three different α distributions, where α used the following values, 0.1, 1 and 10. Analyses for habitat and cell type used α = 1. For multi-state characters (cell diameter) the bias parameter between states is specified as simply 1/κ, where k is the number of states. The overall rate of substitution for both of these classes is a branch length multiplier drawn from a prior gamma distribution. Supplementary Table S2 contains the characters states used for the three characters studied here.

## Additional Information

**How to cite this article**: Sánchez-Baracaldo, P. Origin of marine planktonic cyanobacteria. *Sci. Rep.* **5**, 17418; doi: 10.1038/srep17418 (2015).

## Supplementary Material

Supplementary Information

## Figures and Tables

**Table 1:** Posterior age estimates in Myr using a Bayesian approach.

Node ID	Max root age	MCMCtree				Phylobayes	
		RNA		Protein		RNA	Protein
		HKY85	REV	LG	REV	CAT-GTR	CAT-GTR
1	2,700	2,595 (2,387, 2,699)	2,594 (2,384, 2,700)	2,638 (2,507, 2,712)	2,638 (2,504, 2,711)	2,683 (2,512, 2,873)	2,631 (2,499, 2,705)
	3,000	2,879 (2,609, 3,016)	2,880 (2,614, 3,016)	2,942 (2,787, 3,045)	2,942 (2,788, 3,047)	2,812 (2,626, 3,013)	2,830 (2,631, 3,040)
2	2,700	2,351 (2,136, 2,506)	2,351 (2,136, 2,508)	2,383 (2,235, 2,5013)	2,361 (2,210, 2,483)	2,407 (2,254, 2,583)	2,460 (2,236, 2,589)
	3,000	2,604 (2,336, 2,787)	2,605 (2,342, 2,788)	2,651 (2,472, 2,791)	2,654 (2,486, 2,796)	2,559 (2,390, 2,751)	2.665 (2,486, 2,863)
3	2,700	1,907 (1,659, 2,122)	1,907 (1,666, 2,122)	1,996 (1,836, 2,141)	1,983 (1,820, 2,126)	1,977 (1,602, 2,267)	2,154 (1,940, 2,335)
	3,000	2,108 (1,821, 2,351)	2,109 (1,823, 2,354)	2,213 (2,031, 2,380)	2,221 (2,045, 2,385)	2,091 (1,636, 2,403)	2,338 (2,156, 2,553)
4	2,700	1,972 (1,769, 2,148)	1,973 (1,764, 2,148)	1,894 (1,734, 2,044)	1,904 (1,740, 2,054)	2,090 (1,980, 2,223)	2,126 (1,901, 2,326)
	3,000	2,180 (1,933, 2,374)	2,182 (1,938, 2,377)	2,097 (1,917, 2,2697)	2,103 (1,923, 2,273)	2,386 (2,230, 2,564)	2,325 (2,167, 2,531)
5	2,700	1,421 (1,191, 1,658)	1,4210 (1,197, 1,654)	1,527 (1,377, 1,669)	1,536 (1,387, 1,680)	1,471 (1,053, 1,863)	1,849 (1,618, 2,087)
	3,000	1,569 (1,309, 1,838)	1,570 (1,312, 1,835)	1,874 (1,701, 2,041)	1,882 (1,708, 2,050)	1,554 (1,146, 1,951)	1,994 (1,767, 2,225)
6	2,700	1,345 (1,098, 1,600)	1,345 (1,106, 1,602)	1,372 (1,199, 1,572)	1,409 (1,223, 1,605)	1,350 (784, 1,810)	1,062 (653, 1,692)
	3,000	1,485 (1,211, 1,770)	1,486 (1,210, 1,774)	1,512 (1,317, 1,728)	1,518 (1,3257, 1,737)	1,407 (860, 1,845)	1,487 (873, 2,052)
7	2,700	1,305 (1,128, 1,478)	1,306 (1,128, 1,481)	1,104(979, 1,232)	1,109 (983, 1,238)	1,267 (927, 1,554)	1,047 (709, 1,379)
	3,000	1,437 (1,237, 1,617)	1,439 (1,239, 1,618)	1,150 (1,008, 1,299)	1,153 (1,013, 1,293)	1,330 (1,018, 1,561)	1,190 (779, 1,603)
8	2,700	602 (448, 781)	603 (446, 781)	587 (482, 703)	582 (477, 695)	405 (232, 701)	469 (298, 774)
	3,000	661 (490, 860)	664 (491, 865)	651 (534, 784)	653 (534, 778)	444 (259, 740)	506 (310, 840)
9	2,700	408 (321, 507)	409 (321, 508)	757 (666, 853)	757 (664, 856)	287 (180, 442)	659 (494, 860)
	3,000	452 (354, 561)	452 (355, 561)	841 (740, 948)	846 (743, 949)	299 (202, 446)	688 (553, 887)
10	2,700	270 (195, 365)	270 (194, 364)	550 (454, 654)	563 (465, 671)	188 (105, 329)	560 (316, 841)
	3,000	301 (215, 405)	301 (216, 405)	612 (507, 726)	614 (511, 728)	193 (114, 318)	591 (354, 865)

Node ID corresponds to those shown in Fig. 1. In MCMCtree and Phylobayes, age estimates are given for analyses under the independent rates model^33^ for both RNA and Proteins. In MCMCtree, two separate models of evolution were implemented for RNA and two for proteins. In Phylobayes the CAT-GTR model was implemented for RNA and proteins. Two separate analyses were performed setting the root with a maximum age at 2,700 Myr^63^ and at 3,000 Myr^64^, all analyses implemented a minimum age of 2,320 Myr^2^. Values in parenthesis correspond to posterior 95% confidence intervals associated with median age estimates.
