# Supplementary material for: Origin of marine planktonic cyanobacteria
Source: Sci Rep. 2015 Dec 1;5:17418. doi: 10.1038/srep17418 (PMC4665016; doi:10.1038/srep17418)
Supplement: Supplementary Information [file srep17418-s1.pdf]

Origin of marine planktonic cyanobacteria

Supplementary Information

Patricia Sánchez-Baracaldo[1]

[1]School of Geographical Sciences, University of Bristol, Bristol BS8 1SS, UK

and requests for materials should be addressed to (p.sánchez-baracaldo@bristol.ac.uk)

Materials and Methods

Bayesian divergence time estimation

MCMCtree: For each gene, branch lengths were estimated in BASEML[1] and CODEML[1]. For nucleotides, I tested three of models of nucleotide evolution (HKY85, GTR) and found that age estimates are congruent across models of evolution (Table 1). For proteins, I tested two of models of protein evolution (LG and REV) with age estimates being congruent between both models (Table 1). I used 1 billion years per unit time for all analyses. The substitution rate was calculated independently for each gene in BASEML[1] for nucleotides and in CODEML[1] for proteins (Supplementary Table S1) and an average of all substitution rates was used depending on the analyses. For proteins and nucleotides each gene was treated (Table 1) as separate loci for which the same substitution model is used, but different parameters were assigned and estimated for each partition. The gamma prior used to describe how variable rates are across branches was specified as follows *G* (α and β); the priors used were for proteins G (1, 10.6) and G (1, 12.5) for nucleotides. The mean and standard deviation was specified as m =α/β. The gamma priors for the substitution model parameters κ (transition/transversion rate ratio) and α (gamma shape parameter for variable rates among sites) were all specified by gamma distributions. Respective means and standard deviations were (6, 2) for κ and (1, 1) for α. For all analyses, I used fixed values for the birth-death process $\lambda = \mu = 1$ and $\rho = 0$. Test runs were performed to determine the number of iterations, the burn-in and the sample frequency. All analyses were performed twice to ensure convergence of the MCMC, however only one analysis is reported. For all age calibrations, both minimum and maximum bounds were soft and specified by uniform distributions between the maximum/minimum time constraints with 2.5% tail probabilities above/below these limits allowing for molecular data to correct for conflicting fossil information[1]. Priors on divergence times were assessed by running chains without data to examine prior intervals in relation to expected divergence times. To check whether analyses had converged I used Tracer v1.6[2].

Phylobayes: Additional molecular clock analyses were performed in Phylobayes 3.3b[3] using the same data sets described above. I implemented the Uncorrelated Gamma Multipliers (UGM) model[4] as this model seems to fit better cyanobacteria data sets based on Bayes Factors as previously shown[5]. Because the large data sets analyzed both proteins (6,187 sites) and nucleotides (4,555 sites), I implemented the CAT-GT replacement model as recommended in the manual[3]. I used a birth-death prior on divergence times. I also ran two independent experiments with permissive gamma distributed root priors that allowed a 95% credibility interval of the root node to range between 2,320-2,700 Mya and 2,320-3,000 Mya. I treated all calibration points as soft to allow for 2.5 % on each side for an upper and lower bound.

Supplementary Table S1: Proteins and RNA used to estimate substitution rates and age divergences.

Gene abbreviation and name	Length of alignment	Substitution rate per billion years
Protein sequences		
AtpA, ATP Synthase CF1 Alpha Chain	518	0.085614
AtpB, ATP Synthase F1 Subcomplex Subunit Beta	484	0.068306
AtpH, ATP Synthase CF0 C Chain	82	0.074095
L1 Ribosomal Protein Translation (RplA),	242	0.113598
L4 Ribosomal Protein Translation (RplD)	219	0.163141
L5 Ribosomal Protein Translation (RplE)	182	0.100793
NdhH, NADH Dehydrogenase Subunit 7	395	0.079125
PetB, Cytochrome b6	222	0.055031
PetD, Cytochrome b6-f Complex Subunit IV	161	0.097252
PsaA, Photosystem I p700 Apoprotein A1	857	0.090560
PsbE, Cytochrome b559 Alpha Chain	82	0.160873
RbcL, Ribulose Bisphosphate Carboxylase-Oxygenase	483	0.059598
S10 Ribosomal Protein Translation (RpsJ)	105	0.038472
S13 Ribosomal Protein Translation (RpsM)	117	0.130683
S19 Ribosomal Protein Translation (RpsS)	96	0.077682
SecY, Pre-protein Translocase	123	0.132346
TufA, Elongation Factor Tu	411	0.057311
Ycf3, Photosystem I Assembly Protein	201	0.139499
Nucleotide sequences		
LSU, Large Subunit Ribosomal RNA	3002	0.077336
SSU, Small Subunit Ribosomal RNA	1540	0.081187

Supplementary Table S2. Character states for evolution of morphology, cell diameter and habitat.

**Major Clades and Taxa**	**Morphology**	**Cell Diameter**	**Habitat**
**Macrocyanobacteria**			
Anabaena cylindrica PCC7122	1	2	0
Anabaena sp. 90	1	3	0
Anabaena sp. PCC7108	1	2	0
Anabaena variabilis ATCC29413	1	3	0
Arthrospira maxima CS328	1	3	1
Arthrospira platensis str. Paraca	1	3	1
Arthrospira sp. PCC8005	1	3	1
Calothrix sp. PCC6303	1	3	0
Calothrix sp. PCC7103	1	3	0
Calothrix sp. PCC7507	1	3	0
Chamaesiphon minutus PCC6605	1	1	0
Chroococcidiopsis thermalis PCC7203	0	3	0
Crinalium epipsammum PCC9333	1	2	1
Crocosphaera watsonii WH0003	0	2	1
Crocosphaera watsonii WH8501	0	2	1
Cyanobacterium aponinum PCC10605	1	1	1
Cyanobacterium stanieri PCC7202	1	1	1
Cyanobacterium UCYNA	0	1	1
Cyanobacterium UCYNA2	0	1	1
Cyanothece sp. ATCC51142	0	2	1
Cyanothece sp. ATCC51472	0	2	1
Cyanothece sp. CCY0110	0	2	1
Cyanothece sp. PCC7424	0	2	0
Cyanothece sp. PCC7822	0	2	0
Cyanothece sp. PCC8801	0	2	0
Cyanothece sp. PCC8802	0	2	0
Cylindrospermopsis raciborskii CS505	1	2	0
Cylindrospermum stagnale PCC7417	1	3	0
Dactylococcopsis salina PCC8305	1	3	1
Fischerella muscicola PCC7414	1	3	0
Fischerella muscicola SAG14271	1	3	0
Fischerella sp. JSC11	1	3	0
Fischerella sp. PCC9339	1	3	0
Fischerella thermalis PCC7521	1	3	0
Geitlerinema sp. PCC7105	1	1	0
Geitlerinema sp. PCC7407	1	0	0
Geminocystis herdmanii PCC6308	1	2	0
Gloeocapsa sp. PCC7428	0	2	1
Halothece sp. PCC7418	1	3	1
Leptolyngbya boryana PCC6306	1	1	0
Leptolyngbya sp. PCC7376	1	0	1
Lyngbya majuscula 3L	1	3	1
Lyngbya sp. PCC8106	1	?	1
Mastigocladopsis repens PCC10914	1	2	0
Microchaete sp. PCC7126	1	3	0
Microcoleus chthonoplastes PCC7420	1	2	1
Microcoleus sp. PCC7113	1	2	0
Microcoleus vaginatus FGP2	1	?	0
Microcystis aeruginosa NIES843	0	2	0
Microcystis aeruginosa PCC9443	0	2	0

Microcystis aeruginosa PCC9806	0	2	0
Nodularia spumigena CCY9414	1	3	1
North Pacific *Trichodesmium*	1	3	1
Nostoc azollae 0708	1	3	0
Nostoc punctiforme PCC73102	1	3	0
Nostoc sp. PCC7107	1	3	0
Nostoc sp. PCC7120	1	2	0
Nostoc sp. PCC7524	1	3	0
Oscillatoria acuminata PCC6304	1	2	0
Oscillatoria formosa PCC6407	1	2	0
Oscillatoria nigro viridis PCC7112	1	3	0
Oscillatoria sp. PCC10802	1	?	0
Oscillatoria sp. PCC6506	1	2	0
Oscillatoriales cyanobacterium JSC12	1	1	0
Pleurocapsa sp. PCC7319	1	3	1
Pleurocapsa sp. PCC7327	1	3	0
Prochloron didemni P1 Palau	0	3	1
Prochloron didemni P2 Fiji	0	3	1
Prochloron didemni P3 Solomon	0	3	1
Prochloron didemni P4 Papua New Guinea	0	3	1
Raphidiopsis brookii D9	1	?	0
Richelia intracellularis HH01	1	3	1
Richelia intracellularis HM01	1	3	1
Rivularia sp. PCC7116	1	2	1
Spirulina subsalsa PCC9445	1	2	1
Stanieria cyanosphaera PCC7437	0	3	0
Synechococcus sp. PCC	0	0	1
Synechocystis sp. PCC6803	0	1	0
Synechocystis sp. PCC7509	0	2	0
Trichodesmium erythraeum IMS101	1	3	1
Xenococcus sp. PCC7305	0	3	1
**Microcyanobacteria**			
Cyanobium gracile PCC6307	0	0	0
Cyanobium sp. PCC7001	0	0	1
Cyanothece sp. PCC7425	0	2	0
Leptolyngbya sp. PCC6406	1	0	0
Leptolyngbya sp. PCC7375	1	0	1
Nodosilinea nodulosa PCC7104	1	0	0
Prochlorococcus marinus str. NATL2A	0	0	1
Prochlorococcus marinus str.AS9601	0	0	1
Prochlorococcus marinus str.MIT9202	0	0	1
Prochlorococcus marinus str.MIT9211	0	0	1
Prochlorococcus marinus str.MIT9215	0	0	1
Prochlorococcus marinus str.MIT9301	0	0	1
Prochlorococcus marinus str.MIT9303	0	0	1
Prochlorococcus marinus str.MIT9312	0	0	1
Prochlorococcus marinus str.MIT9313	0	0	1
Prochlorococcus marinus str.MIT9515	0	0	1
Prochlorococcus marinus str.NATL1A	0	0	1
Prochlorococcus marinus subsp. marinus str. CCMP1375	0	0	1
Prochlorococcus marinus subsp. pastoris str. CCMP1986	0	0	1
Prochlorothrix hollandica PCC9006	0	0	0

Synechococcus elongatus PCC6301	0	0	0
Synechococcus elongatus PCC7942	0	0	0
Synechococcus sp. BL107	0	0	1
Synechococcus sp. CB0101	0	0	1
Synechococcus sp. CB0205	0	0	1
Synechococcus sp. CC9311	0	0	1
Synechococcus sp. CC9605	0	0	1
Synechococcus sp. CC9902	0	0	1
Synechococcus sp. PCC7335	0	1	1
Synechococcus sp. RCC307	0	0	1
Synechococcus sp. RS9916	0	0	1
Synechococcus sp. RS9917	0	0	1
Synechococcus sp. WH5701	0	0	1
Synechococcus sp. WH7803	0	0	1
Synechococcus sp. WH7805	0	0	1
Synechococcus sp. WH8016	0	0	1
Synechococcus sp. WH8102	0	0	1
Synechococcus sp. WH8109	0	0	1
**Basal lineages**			
Acaryochloris marina MBIC11017	0	1	1
Acaryochloris sp. CCMEE5410	0	0	1
Gloeobacter violaceus PCC7421	0	0	0
Pseudanabaena biceps PCC7429	1	1	0
Pseudanabaena sp. PCC6802	1	1	0
Pseudanabaena sp. PCC7367	1	0	1
Synechococcus sp. JA23Bprimea213	0	0	0
Synechococcus sp. JA33Ab	0	0	0
Synechococcus sp. PCC6312	0	0	0
Synechococcus sp. PCC7336	0	0	1
Synechococcus sp. PCC7502	0	0	0
Thermosynechococcus elongatus BP1	0	0	0

Supplementary Figures

Supplementary Figure S1. Genomic tree of cyanobacteria estimated using RaxML (Maximum Likelihood). Bootstrap values are 100 for branches that show no values; values are reported for those that show relatively low values. A total of 131 genomes were analyzed based on 135 proteins and two ribosomal RNAs (SSU and LSU). Bar represents the scale of the branch lengths.

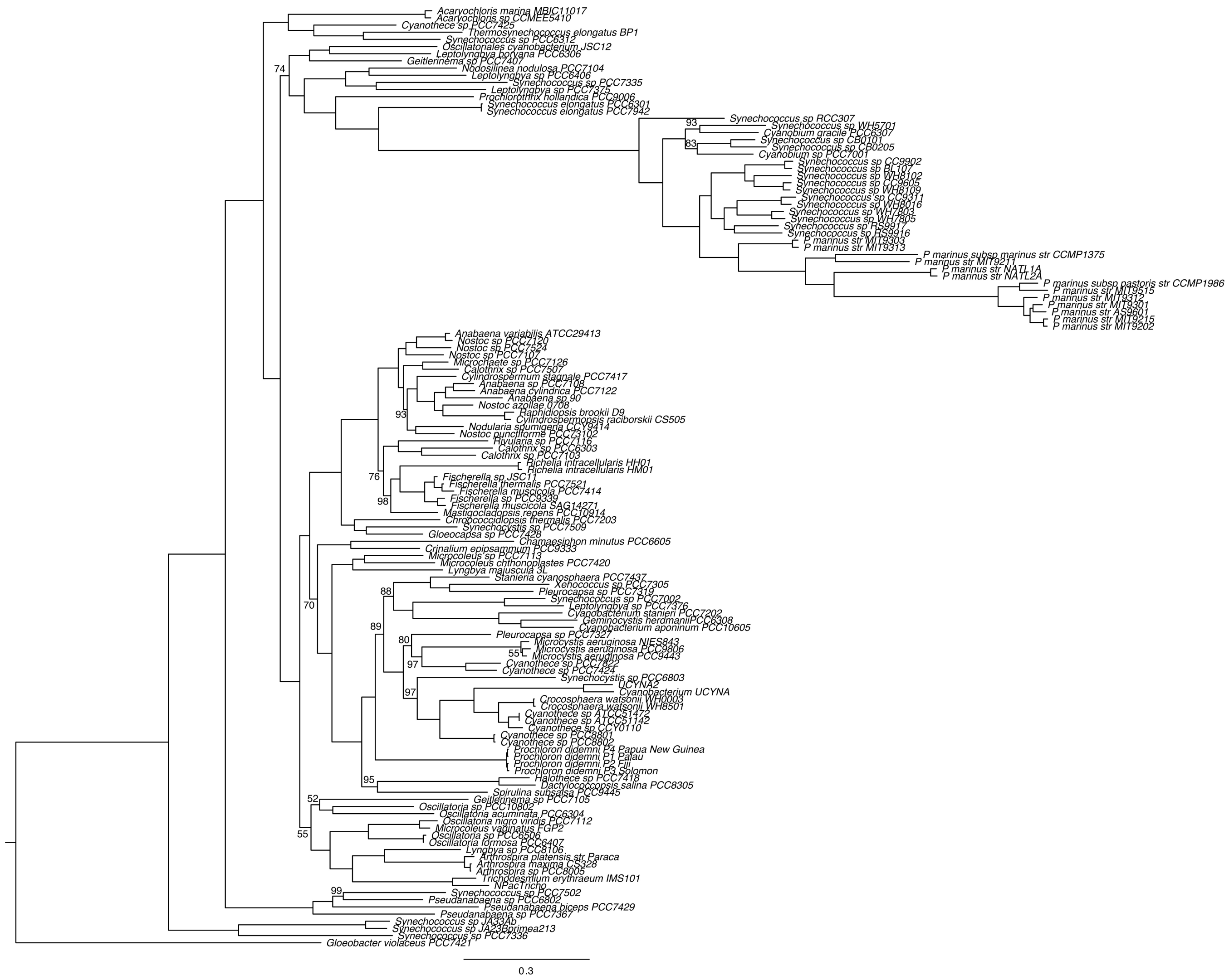

Supplementary Figure S2. Evolution of morphology.

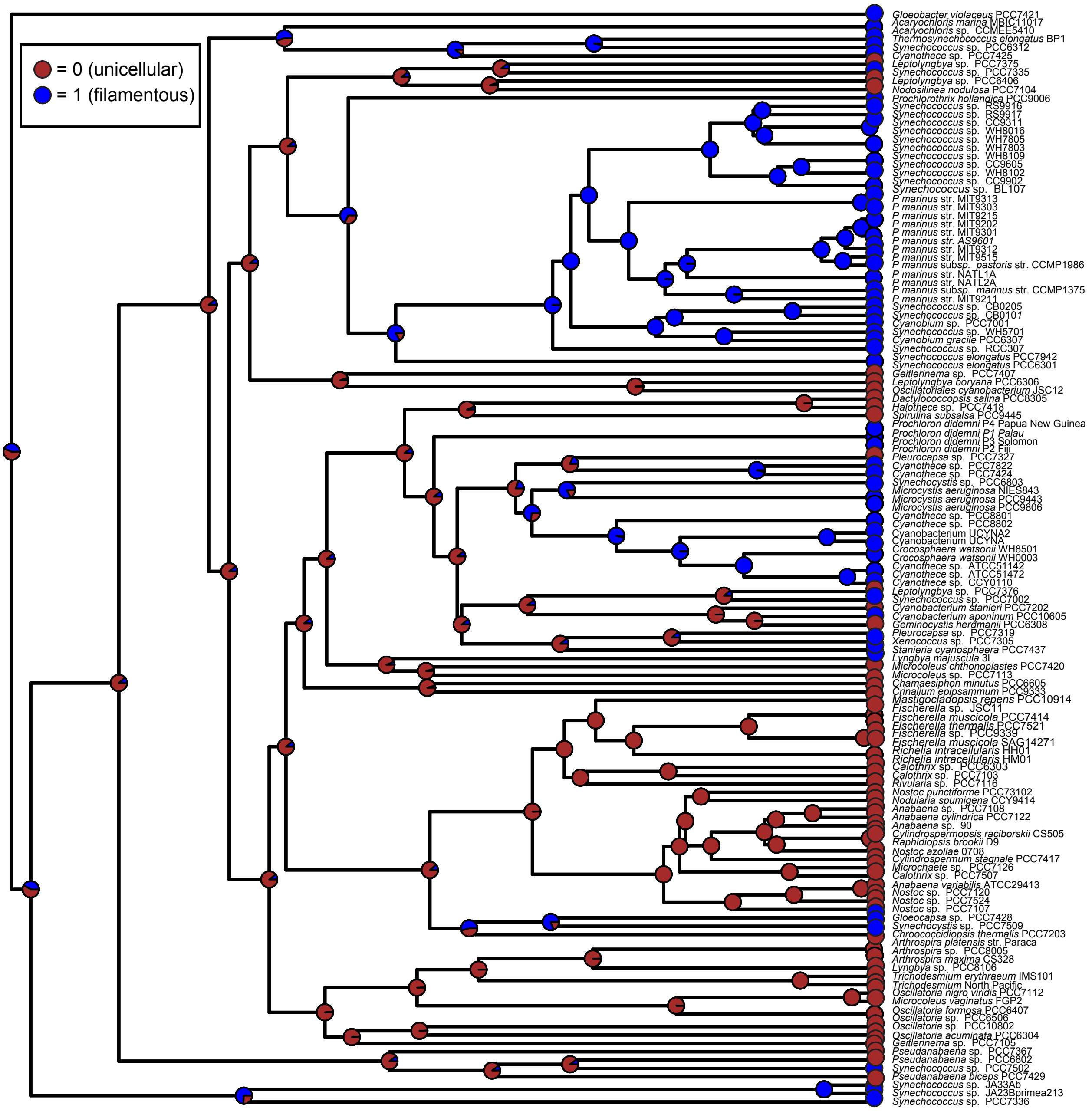

Supplementary Figure S3. Evolution of cell diameter.

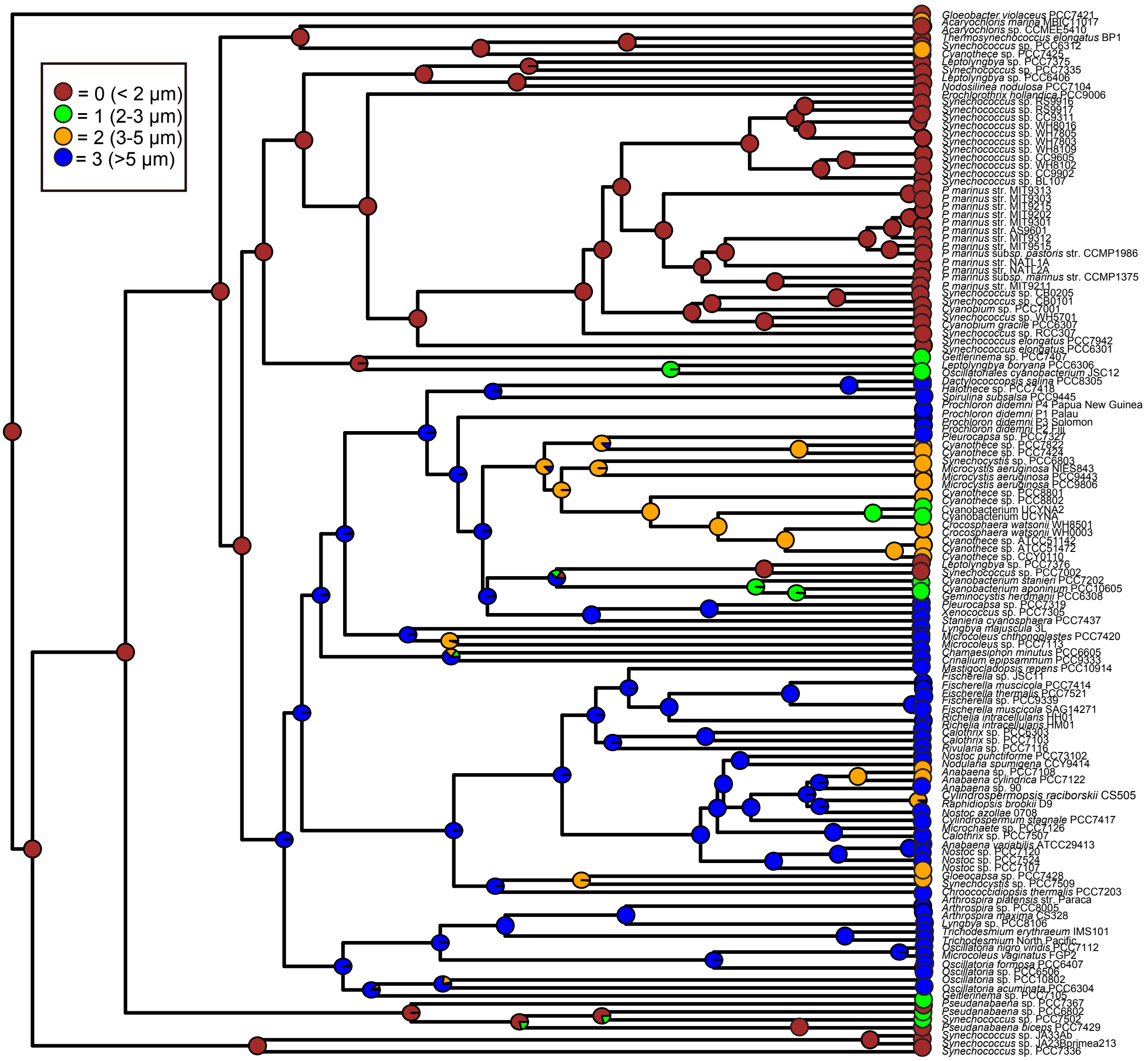

Supplementary Figure S4. Evolution of habitat.

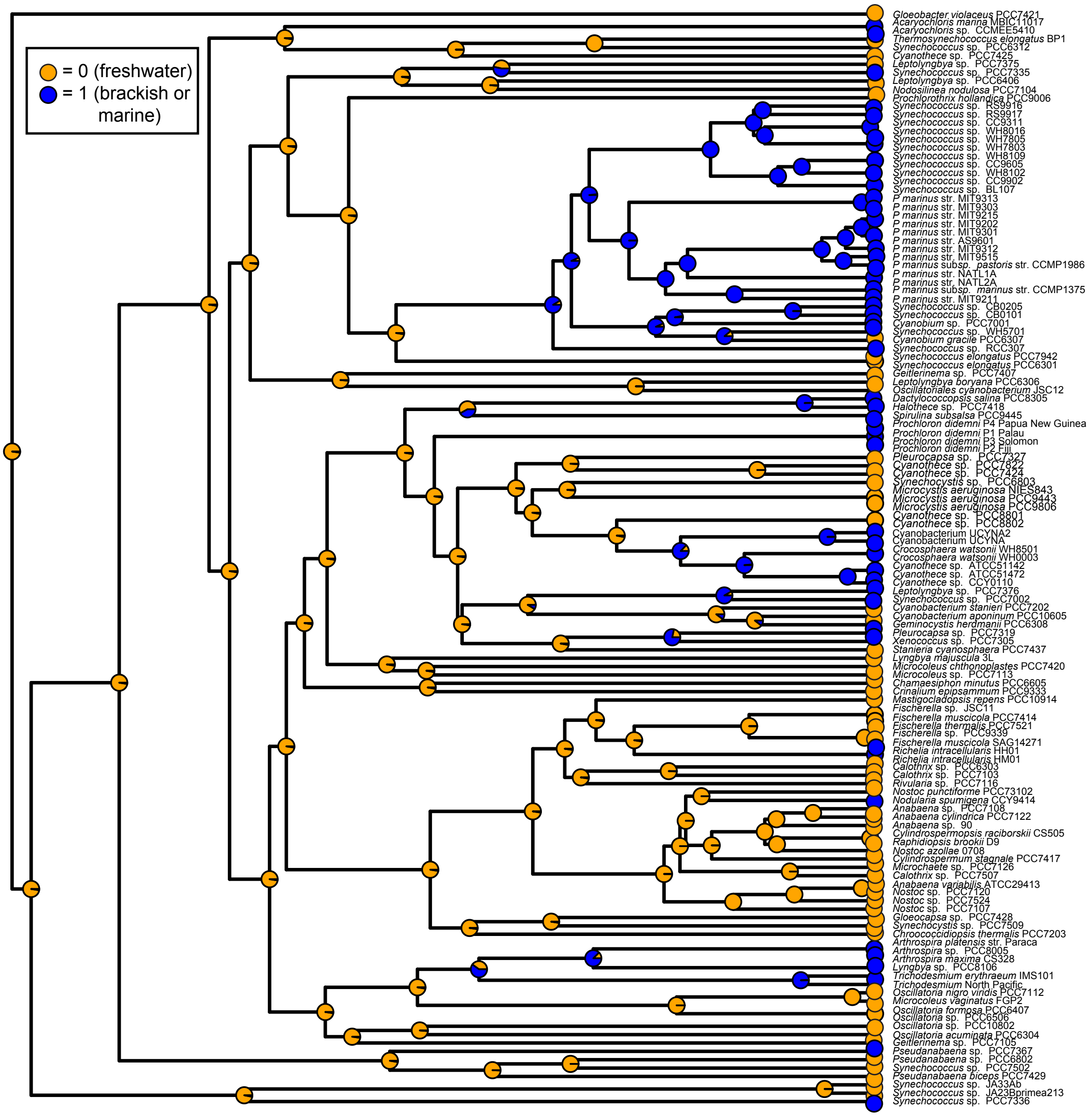

References

1 Yang, Z. PAML 4: Phylogenetic Analysis by Maximum Likelihood. *Mol. Biol. Evol.* **24**, 1586–1591 (2007).

2 Rambaut, A. Suchard, M. A, Xie, D. & Drummond. *Tracer v1. 6.* Edinburgh: University of Edinburgh. Available from http://beast.bio.ed.ac.uk/Tracer. (2014). (Accessed: 6th August 2015)

3 Lartillot N., Lepage T. & Blanquart, S. PhyloBayes 3: a Bayesian software package for phylogenetic reconstruction and molecular dating. *Bioinformatics* **25**, 2286–2288.

4 Drummond, A. J., Ho, S. Y. W., Phillips, M. J. & Rambaut, A. Relaxed Phylogenetics and Dating with Confidence. *Plos Biol.* **4**, e88 (2006).

5 Sánchez-Baracaldo, P., Ridgwell, A. & Raven, J. A. A Neoproterozoic transition in the marine nitrogen cycle. *Curr. Biol.* **24**, 652-657, doi:10.1016/j.cub.2014.01.041 (2014).
